# Low Prevalence of C*fr*-Mediated Linezolid Resistance among Methicillin-Resistant *Staphylococcus aureus* in a Spanish Hospital: Case Report on Linezolid Resistance Acquired during Linezolid Therapy

**DOI:** 10.1371/journal.pone.0059215

**Published:** 2013-03-15

**Authors:** Josep M. Sierra, Mariana Camoez, Fe Tubau, Oriol Gasch, Miquel Pujol, Rogelio Martin, M. Angeles Domínguez

**Affiliations:** 1 Department of Microbiology, Hospital Universitari de Bellvitge. IDIBELL, Department of Pathology and Experimental Therapeutics, Universitat de Barcelona, Barcelona, Spain; 2 Department of Infectious Diseases, Hospital Universitari de Bellvitge. IDIBELL, Department of Pathology and Experimental Therapeutics, Universitat de Barcelona, Barcelona, Spain; Universitätsklinikum Hamburg-Eppendorf, Germany

## Abstract

Linezolid is an effective antimicrobial agent to treat methicillin-resistant *Staphylococcus aureus* (MRSA). Resistance to linezolid due to the *cfr* gene is described worldwide. The present study aimed to analyze the prevalence of the *cfr*–mediated linezolid resistance among MRSA clinical isolates in our area. A very low prevalence of *cfr* mediated linezolid resistance was found: only one bacteremic isolate out of 2 215 screened isolates. The only linezolid resistant isolate arose in a patient, previously colonized by MRSA, following linezolid therapy. Despite the low rate of resistance in our area, ongoing surveillance is advisable to avoid the spread of linezolid resistance.

## Introduction

Linezolid has been introduced into the medical practice to treat Gram-positive infections, especially those related with staphylococcal infections including methicillin resistance *Staphylococcus aureus* (MRSA).

Since the introduction of linezolid in the clinical practice several mechanisms of linezolid-resistance have been described. The major mechanism of resistance is mediated by mutations in the V domain of the 23S rRNA. The most common mutation found is located in the position 2,576 (*E.coli* numbering). Other mutations close to the 2,576 position could also confer linezolid resistance [1], [2]. Due to the presence of multiple copies of the 23SrRNA gene, a relationship between the number of mutated genes and the level of resistance is well established, and known as “gene dosage” [3]. Another mechanism involved in linezolid resistance is the modification of ribosomal proteins L3 and L4 encoded by *rplC* and *rplD* genes, respectively. Some of the mutations found in these genes are concurrent with mutations in the V domain of the 23S rRNA [4]. Finally, RNA methylation by two different methyltranferases has been related to linezolid resistance: *RlnM* - a codon insertion in the methyltransferase gene *rlmN* reduces linezolid susceptibility in clinical *S.aureus* [5], and a methyltranferase encoded by the *cfr* gene. The *cfr* gene is mostly plasmid-located [6] and confers cross resistance to phenicols, lincosamides, oxazolidinones, pleromutilines amd streptogramin A (PhLOPS phenotype). Some outbreaks of *crf*-mediated linezolid-resistant strains have been reported in the literature, such as the recent outbreak described in Spain by Morales et al [7]. In this case, the epidemic strain was involved in surgical site infections; ventilator-assisted pneumonia; and primary bacteremia in an intensive care unit, affecting a total of 12 patients.

The objective of our work was to evaluate the prevalence of *cfr* gene among MRSA clinical isolates in Hospital Universitari de Bellvitge (HUB) from 1999 to 2010.

## Materials and Methods

In the HUB 2,215 clinical MRSA isolates from single patients were isolated from 1999 to 2010. Antibiotic susceptibility to cefoxitin, oxacillin, erythromycin, clindamycin, gentamicin, tobramycin, ciprofloxacin, tetracycline, rifampin, chloramphenicol, vancomycin and teicoplanin was studied in all strains by the disc diffusion method, following CLSI guidelines. Isolates resistant to both clindamycin and chloramphenicol, potentially linezolid-resistant, were screened for the *cfr* presence. Susceptibility to linezolid was studied in this group by the disc diffusion method and microdilution (0.06 mg/L to 128 mg/L) according to CLSI guidelines. The presence of *cfr* gene was analyzed by PCR. Two strains carrying the *cfr* gene and previously characterized [8] were used as controls. Linezolid-resistant strains were genotyped by multilocus sequence typing (MLST), following the methodology described by Enrigh *et al*. [9], sequence types (STs) were determined by comparing with the MLST database (www.mlst.net). Staphylococcal Chromosome Cassette *mec* (SCC*mec*) typing and *agr* polymorphism were studied by PCR according to previously published procedures [10], [11].

## Results and Discussion

Linezolid was introduced in the clinical practice in HUB during 2003. Linezolid prescription average in our hospital during the 2004–2010 period was of 0.28 defined daily dose (DDD)/100 patients-days, with a peak of 0.50 DDD/100 patients-days in 2009.

Only 16 MRSA isolates (0.7%) had the clindamycin and chloramphenicol resistance profile. Linezolid MICs were ≤2 mg/L in all but one strain which showed MIC of 8 mg/L and carried the *cfr* gene. Summarized MICs and results are shown in table 1. The linezolid resistant strain was isolated from a blood culture in April 2009 from a 76 year-old man. The patient had been admitted to the intensive care unit (ICU) in February 2009 because of complications derived from a laryngeal cancer surgery performed on December 2008. The patient carried a nasal MRSA on admittance. During March multiple antibiotics were administered, including linezolid over 19 days, to treat a MRSA respiratory low-tract infection. The initial respiratory MRSA isolates as well as the nasal isolate were resistant to erithromycin, clindamycin, gentamycin, tobramycin, ciprofloxacin, rifampin and susceptible to chloramphenicol and linezolid. The linezolid-resistant MRSA strain was isolated, after linezolid administration, in a single central catheter blood culture and subsequently in different respiratory samples, though no specific therapy was adopted. The patient finally died on May 2009, from a cardiac arrest of unknown etiology. The linezolid resistant isolate was also resistant to erithromycin, clindamycin, gentamycin, tobramycin, ciprofloxacin, rifampin and chloramphenicol and belonged to clone ST228-MRSA-SCC*mec*I with *agr* type II. Linezolid susceptible MRSA strains also belonged to clone ST228-MRSA-SCC*mec*I. No further spread of the linezolid resistant strain to other patients was detected.

**Table 1:** Linezolid susceptibility by MIC and disc-diffusion in 16 clindamycin and chloramphenicol resistant MRSA isolates and presence of the *cfr* gene.

Number of strains	Linezolid MIC (mg/L)	Linezolid inhibition zone (mm)	*cfr* gene
15	1–2	29–32	−
1	8	25	+
Control strains (n = 2)	8–16	26	+

Other reports worldwide described the presence of the *cfr* gene in different *S.aureus* genotypes either of community, ST8-MRSA-SCC*mec*IV and ST398-MRSA-SCC*mec*V [12], [13], or nosocomial origin, ST125-MRSA-SCC*mec*IVc [8]. Thus, the presence of this mechanism of resistance in different *S.aureus* genotypes could potentially be easily spread worldwide due to its plasmid location. In the case reported here, we did not perform additional studies on coagulase-negative *staphylococci*, isolated from the same patient or from other patients admitted to the ICU, that could play a role as potential reservoir of the *cfr* gene for MRSA strains.

In our series, overall linezolid resistance mediated by the *cfr* gene is very low in this period (0.05%; 1/2,215), similar to other surveillance studies [14]. Among clindamycin and chloramphenicol resistant strains, *cfr*-mediated linezolid resistance was 6.25% (1/16). Kerenberg et al [15] found the *cfr* gene in 3% of chloramphenicol-resistant strains of *Staphylococcus spp*. of animal and human origin.

The disc-diffusion technique is not suitable to recognize linezolid resistance mediated by the *cfr* gene with the current CLSI or EUCAST breakpoints. In our experience, staphylococcal isolates exhibiting a resistance phenotype to clindamycin, chloramphenicol and linezolid could suggest for a possible presence of *cfr*. However, further molecular investigations are needed due to the low *cfr* prevalence (6.25%) observed in this studied population. Other linezolid resistance mechanisms, non *cfr*-mediated, are possible, but they do not necessarily involve clindamycin and chloramphenicol resistance.
